# Ultrasound Guided Intraoperative Wire Localization Under General Anesthesia in Breast-Conserving Surgery

**DOI:** 10.7759/cureus.41662

**Published:** 2023-07-10

**Authors:** Artin Vartanian, Paraskevi V Papas, Jesus E Guarecuco Castillo, Michael Sistare, Mohammed M Masri

**Affiliations:** 1 General Surgery, St. George's University School of Medicine, St. George's, GRD; 2 General Surgery, Larkin Community Hospital, South Miami, USA

**Keywords:** intraoperative ultrasounds, breast cancer, tumor localization, breast cancer treatment, ultrasound guided imaging, breast-conserving surgery, wire localization

## Abstract

Breast-conserving surgery (BCS) is becoming an increasingly preferred surgical technique for treating breast cancer. For the last several decades, using a preoperative wire placed by a radiologist has been the gold standard to help guide surgeons to excise a suspicious mass. In recent years, there has been an increasing focus on using surgeon-performed intraoperative ultrasound (IOUS) during breast-conserving therapy, suggesting improved cosmetic outcomes and a decreased need for re-excision. However, studies have also highlighted that ultrasound may be uncomfortable for surgeons who have become most familiar with a wire-localization technique. Wire localization and intraoperative ultrasound are valuable tools that can improve the accuracy of tumor localization and reduce the need for re-excision. We present a 45-year-old female with a right breast mass, measuring breast imaging reporting and data system (BIRADS) 4A on preoperative ultrasound. Intraoperative wire-localization was performed by the surgeon utilizing ultrasound guidance. The right breast lesion was successfully excised with negative margins. The patient was discharged home and recovered well. Surgeon-performed intraoperative ultrasound can be combined with surgeon-performed wire localization to reduce the need for re-excision surgery and allow the surgeon to retain the familiarity of utilizing a gold-standard technique. Further research is needed to determine the optimal use of surgeon-performed IOUS and wire-localization, and its impact on long-term outcomes.

## Introduction

Breast cancer is the most common malignancy in women within the United States, with an estimated 297,790 new cases of invasive breast cancer expected in 2023 [1]. Over the last several decades, advancements in medicine, including screening mammograms, have increased the number of women diagnosed with early-stage breast cancer. Surgeons frequently strive to minimize the removal of healthy tissue while removing non-palpable lesions. The number of breast-conserving surgeries performed annually in the United States today exceeds the number of mastectomies performed [2].

Soon after screening mammograms were implemented in the 1970s, a wire-localization technique was discovered, which allowed for preoperative localization of a mass visualized on mammography. This wire technique enabled surgeons to remove a suspicious mass with greater precision and less healthy tissue removal, allowing for more significant breast conservation [3]. An important emphasis has been placed on improving the accuracy of breast-conserving surgery, specifically aiming for a higher rate of negative margins and, thus, lower future recurrence. Traditionally, wire-guided localization involves a sonographic technique before surgery, requiring two separate procedures, one for the wire localization placement and another for the operative removal of the lesion [3]. This requires the involvement of multiple departments, including surgery and radiology. This can increase patient discomfort and cost and may come with scheduling difficulties, delays, and increased time to treatment [4]. We present a case of a patient who underwent ultrasound-guided intraoperative wire localization under general anesthesia to remove a right breast lesion.

## Case presentation

**Figure 1:**
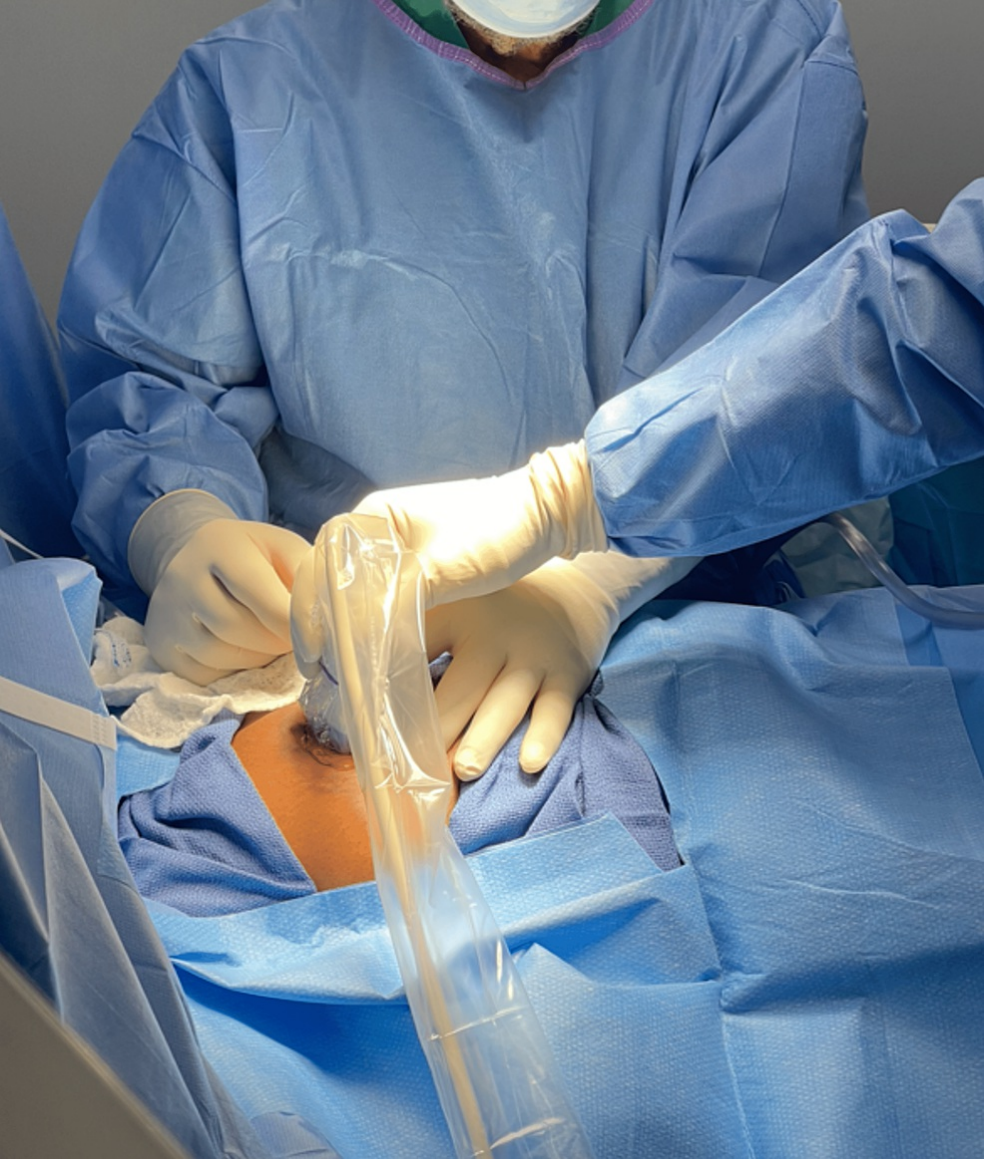
Ultrasound-guided localization of breast lesion.

**Figure 2:**
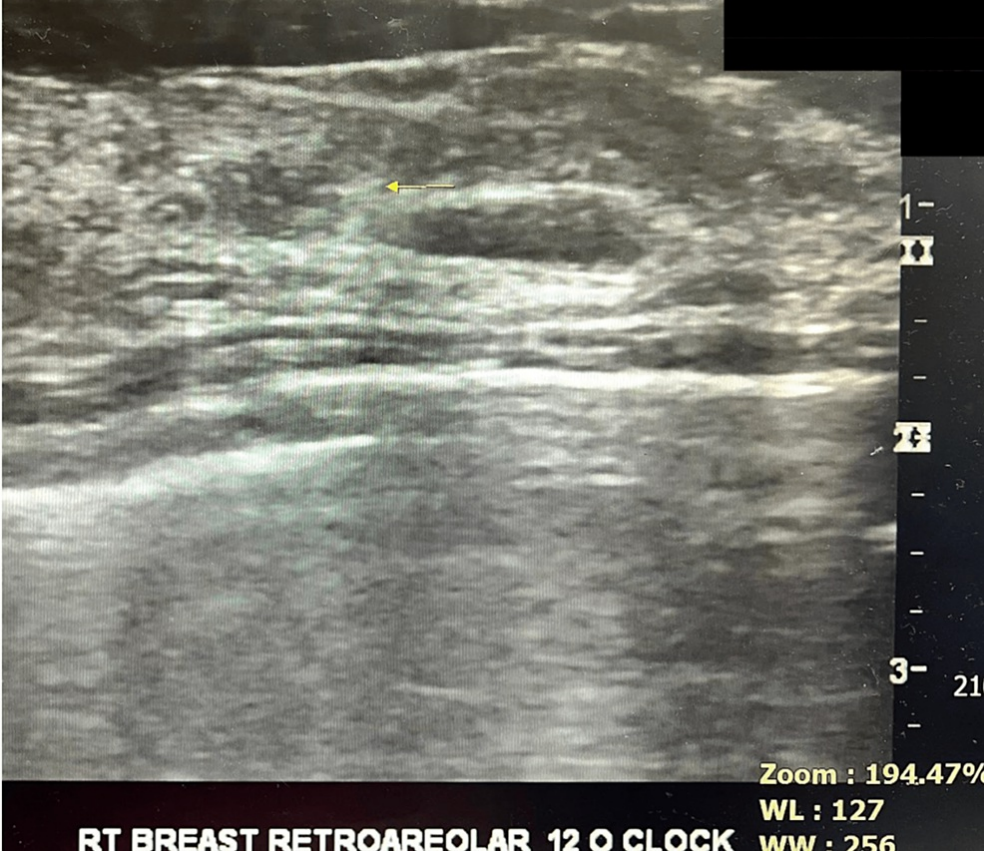
Identification of breast lesion for wire placement.

**Figure 3:**
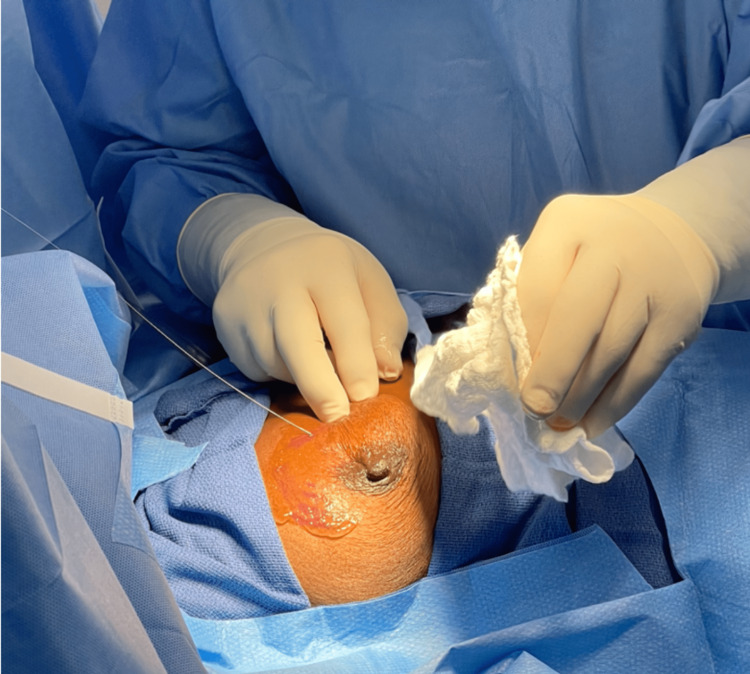
Advancing wire into breast lesion.

**Figure 4:**
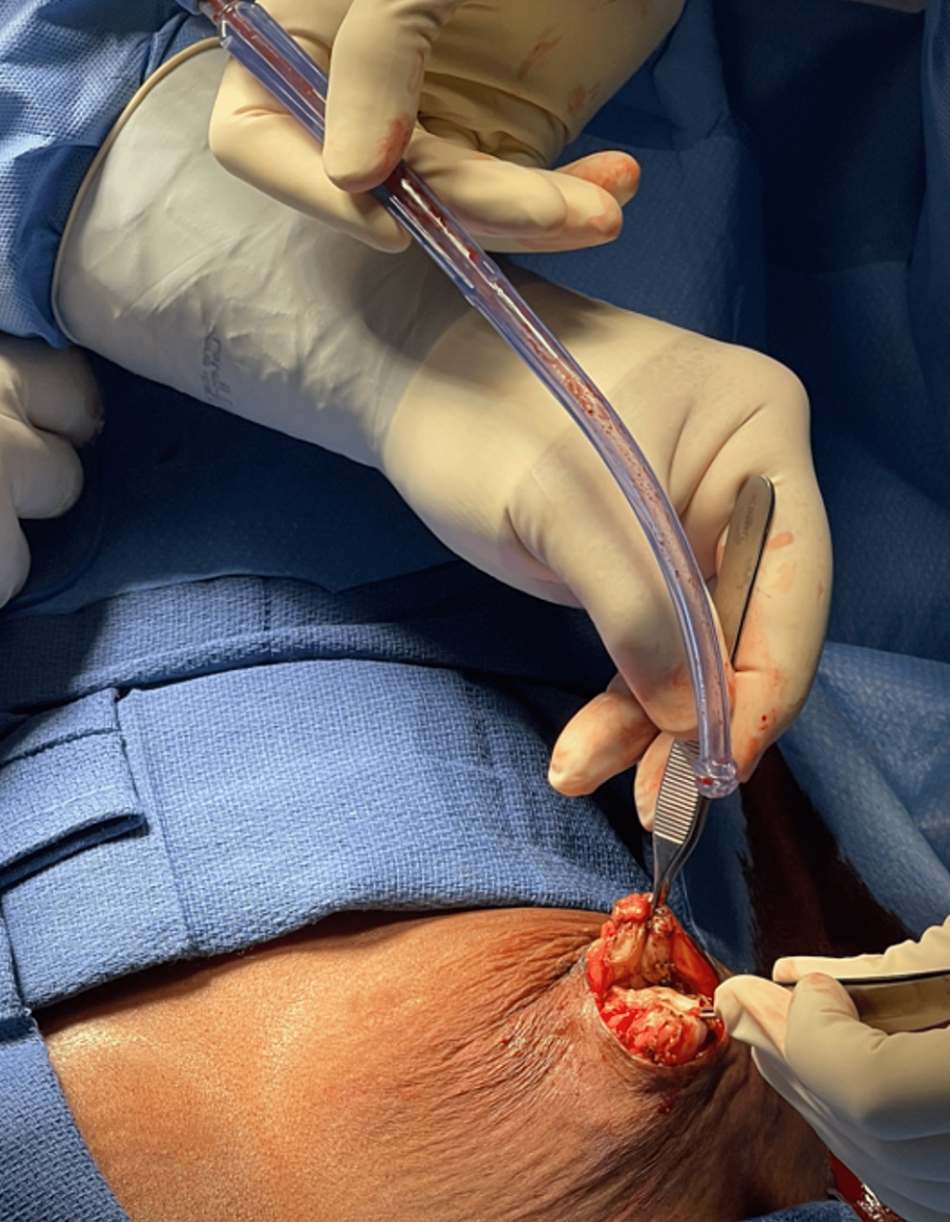
Removal of breast mass.

**Figure 5:**
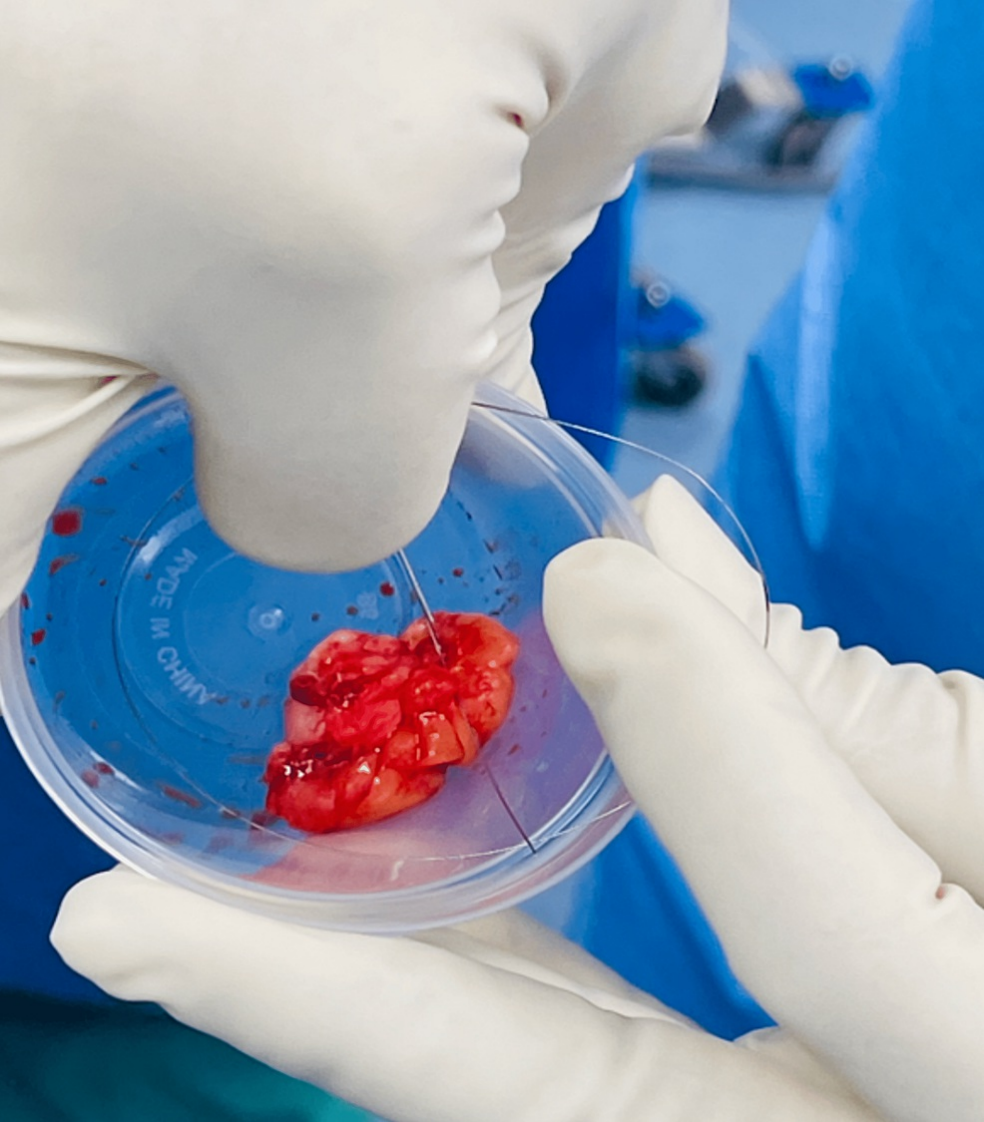
Breast mass with retained wire.

A 45-year-old Black female presented with a non-palpable supra-areolar right breast mass measured as breast imaging reporting and data system (BIRADS) 4A on ultrasound preoperatively. In the preoperative area, the breast lesion was confirmed on ultrasound, which was described as x4 and measured approximately 7 mm in length and intraductal. The patient was intubated, and general anesthesia was induced. Intraoperatively, we visualized the lesion on ultrasound (Figure 1). We then placed a localization wire into the lesion under direct ultrasound guidance (Figures 2, 3). A supra-areolar incision was made at the margin from the 10 o’clock to 2 o’clock position. The previously placed wire guided the incision down until an area of indurated tissue was encountered, indicative of the mass seen on ultrasound. The mass was excised with a #10 scalpel blade and sent to pathology as a frozen specimen (Figures 4, 5). Electrocautery was used to cauterize any bleeding after wire placement. The wound was irrigated with sterile saline. We performed local tissue rearrangement by approximating the subcutaneous fat and breast tissue to allow for the best cosmetic option. The dermal layer was closed with interrupted 3-0 vicryl sutures in a buried fashion. The skin was approximated using a standard running subcuticular suture technique with 4-0 monocryl sutures. The skin overlying the breast incision was injected with 10 mL of 0.25% marcaine, and steri-strips were placed over the wound. The patient tolerated the procedure well with no perioperative complications and was transferred to the anesthesia team with the patient in stable condition while intubated. After extubation, the patient was discharged home following adequate recovery in the post-anesthesia care unit. Follow-up with the patient in an outpatient clinic revealed no complications. The patient was advised to control pain with per oral (PO) Tylenol and contact the surgical team with further questions or concerns.

Management and outcome

Right breast specimens sent to the pathology department for histological examination included a frozen section and tissue from the lumpectomy. The pathology report indicated the following findings for both specimens: cystically dilated lobular units with calcification and fibrosclerosis. Both specimens were negative for atypia or malignancy. In addition, the frozen section displayed findings consistent with ductal ectasia, whereas the lumpectomy tissue showed results consistent with papilloma. 

The patient was seen and evaluated in an outpatient clinic twelve days following the surgery. Pathology results were reviewed and discussed with the patient. She had no postoperative complaints and was recovering adequately. Given her improving status, she was cleared to resume all activities and return to work three weeks after the surgery. The patient was instructed to follow up at the outpatient clinic in six months if her symptoms remained unchanged.

## Discussion

The discovery of wire-localization in the late 1970s allowed surgeons to excise the suspected lesions while conserving as much breast tissue as possible [3]. This technique has remained the gold standard for several decades but has shortcomings. Various localization methods have obtained negative margins, including palpation-guided, radio-guided, or wire-guided. Our technique aimed to combine an intraoperative ultrasound to guide surgeon-performed wire localization and removal of the breast lesion throughout one procedure under general anesthesia [5,6].

Breast-conserving therapy aims to successfully excise the tumor and achieve negative margins, accurately staging the axilla and preserving the cosmetic appearance of the breast [7]. Traditionally, wire-localization occurred preoperatively, with the involvement of a radiologist. A radiologist reviews imaging of the suspected lesion, confirms the location, and administers a local anesthetic before inserting a wire percutaneously into the breast. Typically, this preoperative wire-localization is done on the day of surgery. A significant drawback to this process is the increased pain and discomfort that may be experienced, as these patients are awake during the wire-localization. Previous literature has reported increased anxiety levels in women undergoing preoperative wire localizations [8]. They often must be mindful and restrict their movement to keep the wire secure until surgery is performed. Migration of the wire after the initial placement is also a feared complication of preoperative wire localization [9]. Often a patient is left feeling uncomfortable not only during the placement of the wire by the radiologist but up until the removal during surgery. This timespan can vary from a few hours to over a day. This variability may further amplify a patient’s anxiety levels. As increased preoperative anxiety has been shown to worsen postoperative outcomes, mitigating such stress benefits the surgeon and the patient [10].

Preoperative wire localization can also be challenging due to workflow logistics, as two separate teams, radiology, and surgery, are involved. Any delay in procedure times, scheduling difficulties, or patient transport can negatively impact the patient by leaving the wire in for greater amounts of time [9]. Potential scheduling difficulties may prolong the time from diagnosis to surgery, which has been shown to lower survival rates. Additionally, the preferred surgical incision site by the surgeon may differ from the wire entry site performed by the radiologist, leading to some restrictions. Previously published literature has compared invasive procedures like wire placements to noninvasive techniques, such as intraoperative ultrasound, iodine radioactive seeds, magnetic seed markers, and radar reflectors [3]. The localization devices, such as radioactive seeds and radar reflectors, may be more costly and expose the patient to allergic reactions or unnecessary radiation exposure. The new alternative approaches to pre-operative wire-localization all involve coordinating other teams, including radiology or nuclear medicine physicists, which can increase procedure time and the cost of patient visits [8]. To alleviate the drawbacks mentioned above, performing wire localization intraoperatively under general anesthesia reduces patient pain and anxiety levels, prevents potential scheduling delays and inter-departmental coordination conflicts, and allows for increased surgeon precision of breast mass excision. These factors contribute to more positive postoperative outcomes. 

The utilization of an intraoperative ultrasound allows for real-time visualization of breast lesions which helps direct the surgeon during the procedure [11]. Several studies have concluded that ultrasound-guided lumpectomy is superior to wire-guided lumpectomy in achieving a higher rate of clear margins [12,13]. On the other hand, studies have shown similar success rates between the two techniques. Whether or not there is a superior method requires greater randomized controlled times and further review. However, each of these techniques used individually presents drawbacks. A team of physicians studied the efficacy of intraoperative ultrasonography-guided wire localization and compared it to conventional preoperative wire localization-guided surgery. Reexcision rates and positive resection margins were equivalent within the two groups, but excision volume was smaller in the group that received the intraoperative ultrasound wire localization by a surgeon [8,14]. This confirmed the potential of surgeon-performed wire localization intraoperatively to be a viable alternative to preoperative wire localization with less patient discomfort and lower cost. 

At our institution, 14 cases to date have been completed utilizing an intraoperative surgeon-performed wire localization under ultrasound guidance. Using ultrasound technology during the operation allowed the surgeon to make real-time observations, and using a wire-localization technique offered familiarity [9]. The technique allows patients to be more comfortable as they are not awake between the wire placement and the mass removal. When a surgeon does the wire localization themselves, they are more likely to be able to place an incision without the wire intervening with the preferred planning. Combined with intraoperative ultrasound, which has been associated with lower rates of positive margins and less tissue excision in some studies, this technique can lead to successful resection with less risk [12]. 

## Conclusions

While both ultrasound-guided lumpectomy and wire-guided localization are successful methods in identifying and excising breast lesions, both have their strengths and limitations. Surgeons have experience with both techniques as wire-localization has been the gold standard for many years, and ultrasound-guided lumpectomy has become an increasingly popular alternative. Combining the two methods has increased benefits for both the surgeon and the patient. By simultaneously employing both methods intraoperatively, surgeons can benefit from direct visualization of the lesion, wire guidance during the excision, and obtaining clear surgical margins while conserving as much healthy breast tissue as possible. Performing ultrasound-guided wire localization intraoperatively and under sedation also benefits the patient by increasing comfort and reducing pain associated with traditional wire localization techniques. Wire-localization and surgeon-performed ultrasound may contribute to better cosmetic outcomes as more breast tissue is conserved, which is associated with increased patient satisfaction. Further studies should be performed to evaluate whether implementing this technique is feasible and beneficial to both the surgeon and the patient. 
